# Platelet Responses After Tapering and Discontinuation of Fostamatinib in Patients with Immune Thrombocytopenia: A Continuation of the Fostasur Study

**DOI:** 10.3390/jcm13216294

**Published:** 2024-10-22

**Authors:** María Eva Mingot-Castellano, Gloria García-Donas, Rosa María Campos-Álvarez, María Carmen Fernández-Sánchez de Mora, Josefa Luis-Navarro, Juan Francisco Domínguez-Rodríguez, María del Mar Nieto-Hernández, Irene Sánchez-Bazán, Maria Yera-Cobo, Rocio Cardesa-Cabrera, Francisco José Jiménez-Gonzalo, Isabel Caparrós-Miranda, Laura Entrena-Ureña, Sergio Jurado Herrera, Dolores Fernández Jiménez, Dana Díaz-Canales, Gloria Moreno-Carrasco, Cristina Calderón-Cabrera, Ramiro José Núñez-Vázquez, Begoña Pedrote-Amador, Reyes Jiménez Bárcenas

**Affiliations:** 1 Hospital Universitario Virgen del Rocío, Instituto de Biomedicina de Sevilla (IBIS/CSIC), Universidad de Sevilla, 41013 Sevilla, Spain; cristinac.calderon.sspa@juntadeandalucia.es (C.C.-C.); ramiroj.nunez.sspa@juntadeandalucia.es (R.J.N.-V.); begona.pedrote.sspa@juantadeandalucia.es (B.P.-A.); 2 Hospital Universitario Virgen Macarena, 41009 Sevilla, Spain; gloria.garciadonas.sspa@juntadeandalucia.es; 3 Hospital Universitario de Jerez de la Frontera, 11405 Cádiz, Spain; rosam.campos.sspa@juntadeandalucia.es; 4 Hospital Universitario Reina Sofía, 14004 Córdoba, Spain; carmen.fernandez.sa.sspa@juntadeandalucia.es; 5 Hospital de Riotinto, 21660 Huelva, Spain; 6 Hospital Universitario Juan Ramón Jiménez, 21005 Huelva, Spain; 7 Complejo Hospitalario de Jaén, 23006 Jaén, Spain; 8 Hospital Regional Universitario de Málaga, 29010 Málaga, Spain; 9 Hospital Puerta del Mar, 11009 Cádiz, Spain; maria.yera.sspa@juntadeandalucia.es; 10 Hospital de la Merced de Osuna, 41640 Sevilla, Spain; rocio.cardesa.sspa@juntadeandalucia.es (R.C.-C.); fjo.jimenez.sspa@juntadeandalucia.es (F.J.J.-G.); 11 Hospital Virgen de la Victoria, 29010 Málaga, Spain; isabels.caparros.sspa@juntadeandalucia.es; 12 Hospital Universitario Virgen de las Nieves, 18014 Granada, Spain; laura.entrena.sspa@juntadeandalucia.es; 13 Hospital Torrecárdenas, 04009 Almería, Spain; sergio.jurado.sspa@juntadeandalucia.es; 14 Hospital Universitario San Cecilio, 18007 Granada, Spain; dolores.fernandez.jimenez.sspa@juntadeandalucia.es; 15 Hospital de la Serranía de Ronda, 29400 Málaga, Spain; dana.diaz.sspa@juntadeandalucia.es (D.D.-C.); gloria.moreno.carrasco.sspa@juntadeandalucia.es (G.M.-C.); reyes.jimenez.sspa@juntadeandalucia.es (R.J.B.)

**Keywords:** tapering, sustained response, ITP, fostamatinib, thrombopoietin analogs, SYK inhibitor, SYK, remission, purpura, thrombocytopenic, idiopathic, diarrhea, hemorrhage, thrombocytopenia

## Abstract

**Background/objectives:** Fostamatinib is a spleen tyrosine kinase (SYK) inhibitor approved for the treatment of adult patients with chronic immune thrombocytopenia (ITP). There is little information about dose tapering and sustained remission after discontinuation in ITP. In this retrospective multicenter study, we evaluated efficacy and safety of fostamatinib in adult patients with ITP before, during, and after tapering/discontinuation (T/D). **Methods:** T/D was performed on subjects who achieved complete platelet response (CR) with progressive, conditional dose reduction every four weeks. **Results:** Sixty-one patients were included from 14 reference centers between October 2021 and May 2023. In subjects that completed T/D (n = 9), the median time from treatment initiation to response was 21 days (IQR: 7.5–42), median time from treatment initiation to CR was 28 days (IQR: 28–42), median time from treatment initiation to the start of tapering was 116 days (IQR: 42–140), and duration of tapering was 112.5 days (IQR: 94.5–191). The median platelet count was 232 × 10^9^/L (IQR: 152–345 × 10^9^/L) at tapering and 190 × 10^9^/L (IQR: 142.5–316.5 × 10^9^/L) at discontinuation. With a median follow-up since discontinuation of 263 days (IQR: 247–313 days), only two patients have relapsed (at 63 and 73 days). Fostamatinib was restarted, achieving a new CR. Platelet counts higher than 100 × 10^9^/L in week 12 were the only positive predictive factors for successful tapering and discontinuation. **Conclusions:** Sustained response in patient with ITP treated with fostamatinib could be developed. The prognostic factors and recommended scheme of tapering still have to be evaluated.

## 1. Introduction

Primary immune thrombocytopenia (ITP) is an acquired autoimmune disease characterized by a low platelet count (<100 × 10^9^/L) and an increased bleeding risk [1,2]. First-line therapy for ITP includes steroids and intravenous immunoglobulin (IVIg). Some patients experience a single episode of severe thrombocytopenia and receive effective treatment that induces remission, while 40–70% of adult patients develop chronic ITP (cITP) and require long-term treatment [3].

For patients that do not respond to steroids or become corticosteroid-dependent, there are several options of therapy with strong supporting evidence, including thrombopoietin receptor agonists (TPO-Ras) and rituximab [1,2,4]. Second-line treatments with strong supporting evidence include fostamatinib, a spleen tyrosine kinase (SYK) inhibitor. The effects of fostamatinib are mediated through the inhibition of signal transduction through B cell receptors and Fc-activating receptors on macrophages [5], which reduces antibody-mediated platelet destruction [6].

Fostamatinib has been approved (by the US Food and Drug Administration in 2018 and the European Medicines Agency in 2020) for the treatment of cITP in adult patients who are refractory to other treatments [7,8]. The FIT1 and FIT2 trials and the open-label extension, FIT3, showed that fostamatinib may achieve a platelet response in around 40% of patients [9,10]. A post hoc analysis of the FIT program described a global response rate of 86–94% in cases of early second-line use of fostamatinib [11]. Real-world evidence confirms these data [12,13,14,15,16].

Sustained response off therapy (SROT) is defined as the possibility to discontinue cITP treatment and maintain a safe platelet count for a prolonged period of time [1,17]. Obtaining an SROT has become a key goal of ITP therapy. Previous studies have demonstrated that an SROT may be achieved after splenectomy (60–70%), rituximab (20–30%), and TPO-Ras (10–30%) [2,4,17,18,19].

TPO-Ras may produce an SROT by modifying the immunologic responses in the bone marrow, making the immune system tolerant to platelets [20,21]. The induction of this immune tolerance may allow the safe discontinuation of TPO-Ras. There is little information on SROT with fostamatinib. At present, there is one published case report describing an SROT of 24 months with fostamatinib in a multi-agent refractory cITP patient [15] and the description of five patients who achieved an SROT in a real-world study of fostamatinib safety and efficacy, without any description of patient characteristics, tapering methodology, or quality response or duration [12].

The Andalusian Group of Congenital Coagulopathies (GACC) recently collected and summarized our experience with fostamatinib in clinical practice in the Fostasur study (NCT06071520) [22]. Now, reflecting that in our clinical practice, TPO-RA tapering is recommended in subjects with a platelet count of 50–100 × 10^9^/L that is stable during 4–6 months without rescue treatment [4]. We have applied a similar strategy to fostamatinib. In this paper, we present an update on the Fostasur study that includes data on tapering, discontinuation, and SROT in our patients.

## 2. Methods

### 2.1. Study Design

In this retrospective multicenter study, adult patients diagnosed with primary ITP (newly diagnosed, persistent, or chronic) who were on fostamatinib treatment were evaluated. Fostamatinib was prescribed either according to the Summary of Product Characteristics [7] or based on the healthcare provider’s clinical judgement in regard to dosage, monitoring, and avoiding contraindicated medications.

Given that an SROT is a goal of ITP therapy and that an SROT has been reported with fostamatinib, a tapering/discontinuation protocol was developed for patients showing complete responses to fostamatinib. The criterion for the selection of patients for tapering/discontinuation (T/D) was a platelet count (PLT) greater than 100 × 10^9^/L for at least 6 months. In patients with platelet counts higher than 250 × 10^9^/L, the dose was reduced, despite experiencing less than 6 months of CR, and this was considered the start of tapering. No other criteria were used for patient selection to enter the T/D protocol. Clinical parameters for patients eligible for T/D were compared with parameters of patients not eligible for T/D. The patient characteristics included dosages, PLT responses, previous treatments, and adverse events (Aes).

All patients treated with fostamatinib at the participating clinics that provided informed consent were evaluated consecutively over the period between October 2021 and May 2023. Patients that were eligible for T/D followed this dose reduction protocol. Starting with a dose of 150 mg every 12 h, the dose was reduced to 150 mg alternating with 100 mg every 12 h, then to 100 mg every 12 h, then 100 mg every 24 h, then 100 mg every 48 h, then 100 mg twice a week, and then discontinued. PLTs were monitored every two weeks, and if the PLT remained above 50 × 10^9^/L during four weeks after a dose reduction, an additional dose decrease was initiated.

The primary objective was to describe the efficacy and safety of fostamatinib in real-world use and to describe the characteristics of the patients eligible for T/D, compare them with patients ineligible for T/D, and to describe the rate of SROT and characteristics of this group of patients. These characteristics included type of ITP and response to fostamatinib.

Phases of ITP and responses to fostamatinib treatment were classified according to international guidelines [1,17]. ITP phases were as follows: newly diagnosed ≤ three months from diagnosis; persistent ITP > three months < 12 months from diagnosis; and chronic ITP > 12 months. Responses to fostamatinib were defined as follows: complete response (CR) PLT ≥ 100 × 10^9^/L, response (R) PLT ≥ 30 × 10^9^/L < 100 × 10^9^/L, and non-response (NR) PLT < 30 × 10^9^/L. CR and R were at least a doubling of the PLT from the baseline and the absence of bleeding events. CR and R were combined to give the overall response (OR).

### 2.2. Study Population

This study’s population consisted of patients treated with fostamatinib for ITP between October 2021 (marketing date in Spain) and May 2023 at participating clinics in the Andalusia region of Spain. Inclusion criteria included patients that were 18 years or older, had a diagnosis of ITP according to clinical practice guidelines [2,17,23], and were being treated with fostamatinib. Patients meeting the selection criteria while on fostamatinib treatment were initiated into the T/D protocol.

Patients were excluded from this study if they had any medical or psychological conditions that prevented them from following normal clinical practice procedures as determined by the investigator. In addition, patients with concomitant defects in hemostasis, a documented history of non-ITP medical conditions that were the underlying cause of their thrombocytopenia, secondary ITP, or a known allergy to fostamatinib or its formulation were excluded.

### 2.3. Variables

The following data were collected: demographics, medical history including the type of ITP, ITP treatment history and fostamatinib dosage, periodic PLT, and need for rescue medication.

Aes were also recorded for all patients. Aes included fostamatinib discontinuation due to lack of effectiveness (PLT level insufficient to prevent significant bleeding). Other Aes included high blood pressure, hepatotoxicity, diarrhea, neutropenia, infections, and any other Aes.

### 2.4. Sample Size

In this study, all patients who met the criteria in the defined study period and who signed the informed consent were included. All patients that met the criterion for T/D followed the T/D protocol. Patients that did not meet the T/D criterion were analyzed for comparison with the T/D group. Assessment of the treatment of ITP in Andalusia indicated that 50–70 patients could be enrolled in this study, based on ITP prevalence and the Andalusian population (~8,600,224 persons) [24].

### 2.5. Analysis Plan

Study data were recorded and stored in a specifically designed database. Discrete variables were summarized as numbers and percentages. Continuous variables were described by their medians (interquartile range [IQR]). The Mann–Whitney U-test was used to compare non-parametrically distributed continuous variables. Spearman’s Rho coefficient was calculated to analyze the correlation between non-parametrically distributed variables. The Fisher exact test was used to compare qualitative variables.

### 2.6. Ethical Considerations

Patients, or their legal representatives, provided written informed consent to participate in this study. Any product-related AE was reported to the Spanish Agency of Medicines and Medical Devices (AEMPS). This study’s protocol was approved by the institutional ethics committee (Comité Ético de Investigación, Provincial de Sevilla: ethics committee code 0169-N-23) on 22 May 2023, and it was conducted in accordance with the 2013 revision of the Declaration of Helsinki. The trial was registered at clinicaltrials.gov (NCT06071520).

## 3. Results

The overall population of fostamatinib-treated ITP patients in this study was 61 patients. The population had a slight preponderance of male patients (54.1%) over female patients (45.9%) and a median age of 59 years (Table 1). The most common co-morbidities in this cohort were hypertension, dyslipidemia, heart disease, obesity, diabetes, and arterial thrombosis (Table 2). The WHO hemorrhagic classification was between zero and one for most of the patients (85.2%), and most had cITP (75.4%). Most of the patient population was heavily pre-treated for ITP, with a median of four prior treatments before they were treated with fostamatinib (range 1–9). The most common treatments prior to fostamatinib in these patients were corticosteroids, intravenous immunoglobulins, and TPO-Ras.

**Figure 1:**
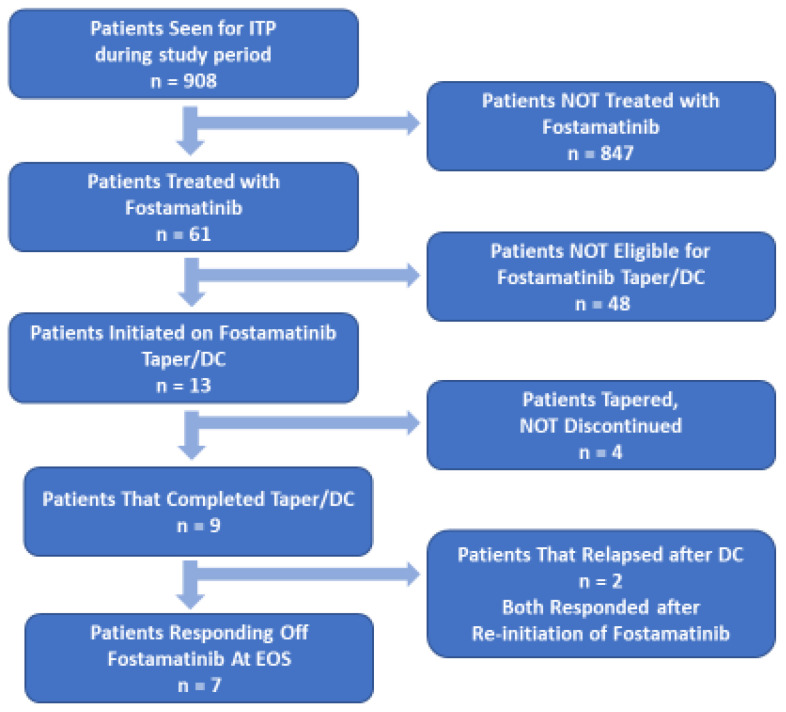
Patient disposition in the Fostasur taper/discontinuation study. Figure 1 shows the disposition of patients from the population of ITP patients treated at participating clinics to those that completed the fostamatinib T/D protocol. Of the population of ITP patients treated at the study clinics during the inclusion period, 61 were treated with fostamatinib. Of the patients treated with fostamatinib, 13 were eligible for the fostamatinib T/D protocol. Nine patients completed the dose taper and were discontinued.

The OR rate to fostamatinib was 72.1% (n = 44; Table 1). Of the responders, 15 (24.6% of fostamatinib-treated patients) had a CR (PLT ≥ 100 × 10^9^/L). Based on the selection criterion described above (PLTs > 100 × 10^9^/L (CR) for at least six months), 13 patients were entered into the T/D protocol. The median PLT in the selected patients at the initiation of the T/D protocol was 232 × 10^9^/L (IQR 152–345 × 10^9^/L). For the nine patients that completed the protocol (fostamatinib was discontinued), the median PLT at the end of T/D was 190 × 10^9^/L (IQR 142.5–316.5 × 10^9^/L). As shown in Figure 1, two of the patients that completed the T/D protocol relapsed. The PLTs at relapse were 9.5 and 11 × 10^9^/L. Both patients had complete responses with reinstatement of fostamatinib at 100 mg every 12 h. No rescue treatment was used between relapse and the second complete response.

The time from the onset of fostamatinib therapy to the initiation of the T/D protocol was a median of 116 days (IQR 38.5–157). Tapering took a median of 112.5 days (IQR 94.5–191), indicating that most patients completed the T/D protocol without delays due to PLTs below the threshold set in the protocol (≤50 × 10^9^/L). For the two patients that relapsed after the T/D protocol, the time from discontinuation to relapse was 63–73 days. The time between the end of the T/D protocol and the last reported follow-up was over six months (median 263 days (IQR: 247–313 days). The median platelet count at the last visit was 156 × 10^9^/L (IQR 135–312 × 10^9^/L) in the T/D and SROT group (7/61 patients) and 112 × 10^9^/L (IQR 47–143.5 × 10^9^/L) in the non-T/D group (31/61 patients).

### 3.1. Comparison Between Eligible and Ineligible Patients for the T/D Protocol

When the patients eligible for the T/D protocol were compared to ineligible patients, the T/D group had a greater proportion of female patients and were slightly younger, but these differences were not statistically significant. Both groups had similar WHO Hemorrhagic Classifications (Table 1). More patients in the non-T/D group required a dose increase (62.5%) than in the T/D group (30.8%). The T/D eligible group had a higher percentage of patients previously treated with rituximab (T/D 38.5% versus non-T/D 18.8%) and splenectomy (T/D 30.8% versus non-T/D 6.3%) than the non-eligible group. A higher proportion of the non-T/D group was refractory or had a suboptimal response to TPO-Ras (60.4% non-T/D versus 38.5% T/D), while the T/D group was more likely to be intolerant to TPO-Ras (T/D 38.5% versus 16.7% non-T/D). These differences between the T/D and the non-T/D groups were not statistically significant.

Comparisons between the T/D and non-T/D groups regarding previous treatments, time since diagnosis, baseline PLTs, starting, responsive and final doses, associated therapies, and need for rescue therapy showed no significant differences between the groups.

**Figure 2:**
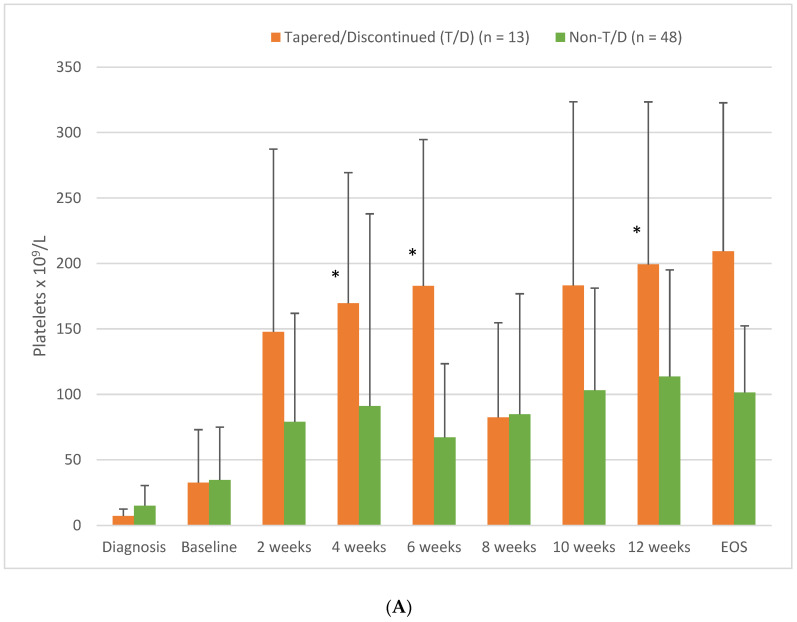

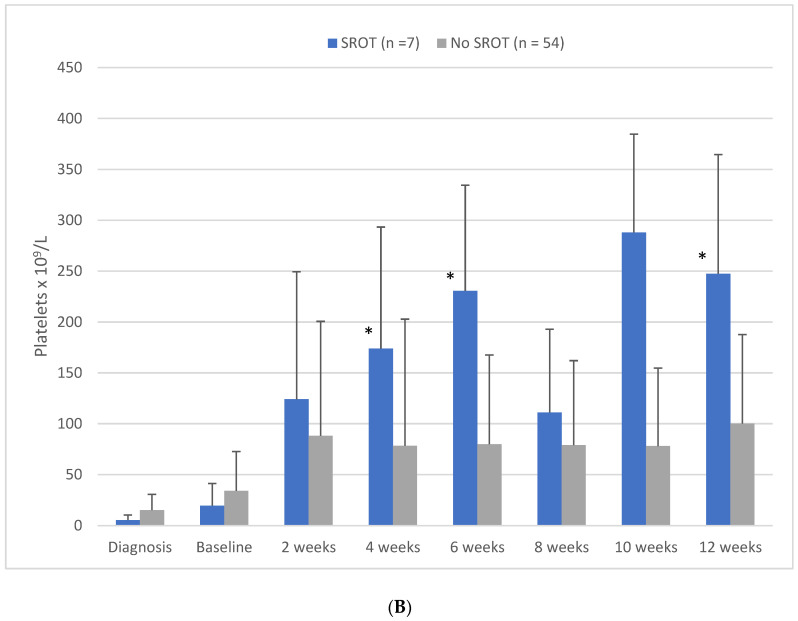
(**A**) Platelet levels in patients with ITP treated with fostamatinib, comparing patients eligible for the tapering/discontinuation (T/D) protocol with those not eligible for T/D. The selection criterion was a PLT > 100 × 10^9^/L for at least six months. Values are mean ± SD. * Significant differences between T/D eligible patients and non-eligible patients (*p* < 0.05). (**B**) Platelet levels in patients with ITP treated with fostamatinib, comparing patients that achieved a sustained response off treatment (SROT) after the tapering/discontinuation protocol (T/D) with those patients that did not. Values are mean ± SD. * Significant differences between SROT patients all other patients (*p* < 0.05).

Some statistically significant differences were observed between the two groups when temporal PLTs were examined. Figure 2A shows the PLTs for both groups (tapered versus non-tapered) over the first 12 weeks of fostamatinib therapy. These data showed that patients eligible for T/D had significantly higher PLTs at weeks 4 (*p* = 0.003), 6 (*p* = 0.012), and 12 (*p* = 0.007) than non-eligible patients. In addition, patients that were eligible for T/D were more likely to have a PLT > 100 × 10^9^/L at 12 weeks than patients that were not eligible for T/D (*p* = 0.011).

### 3.2. Comparison Between Patients That Achieved an SROT and the Rest of Patients

Comparisons were also made between the group that completed the T/D protocol and remained responsive after fostamatinib therapy was discontinued (sustained response off therapy (SROT)) and the other patients treated with fostamatinib in this study. Figure 2B shows that the platelet counts were higher during the first 12 weeks of therapy in patients that developed an SROT. These differences were statistically significant at 4, 6, and 12 weeks of fostamatinib treatment. As noted in Figure 1, of the 13 patients that entered the T/D protocol, four patients had their doses tapered but not discontinued, nine completed the T/D protocol, and two of those nine relapsed. The two patients that relapsed were responsive to re-introduction of fostamatinib therapy.

## 4. Discussion

In ITP patients, SROT has been documented with treatments, including corticosteroids [25], corticosteroids in combination with IVIG [26], eltrombopag [27,28,29,30], and romiplostim [27,31,32]. For SROT with fostamatinib, there is little information. One case report described a 24-month SROT in a multirefractory chronic ITP patient who achieved a complete response with fostamatinib [15]. González-López et al., in their series of 138 patients with ITP treated with fostamatinib, had five patients with an apparent SROT. However, no additional information was provided regarding patient characteristics and follow-up [12]. Finally, the phase 3 randomized, double blind placebo-controlled study of another SYK inhibitor, sovleplenib, in ITP patients did not describe discontinuation or SROT [33].

The Fostasur study was designed retrospectively to describe real-world practice management, efficacy, and safety of fostamatinib in ITP patients in Andalusia. Since a common goal of ITP therapy is to minimize or discontinue therapy while maintaining a clinical response, i.e., SROT [1,2,4,23], a secondary objective of the study was to identify clinical characteristics of patients that achieved an SROT after fostamatinib treatment. This could allow better prediction of which fostamatinib-treated patients would be the best candidates for T/D in routine clinical practice. In this current study, we determined that early responses to fostamatinib may help to predict which patients may be amenable to T/D while maintaining a therapeutic response.

When patients eligible for the T/D protocol were compared with those not eligible, most of the demographic, clinical, and treatment parameters analyzed in this study did not show significant differences between these groups. The only clinical factors that showed a significant difference between the T/D eligible patients versus non-eligible and patients with an SROT versus all other patients were platelet counts at 4, 6, and 12 weeks of fostamatinib treatment (Figure 2A,B). Patients eligible for T/D and with an SROT also had a PLT > 100 × 10^9^/L at 12 weeks of fostamatinib treatment (*p* = 0.007 and *p* = 0.011, respectively) more frequently. These data suggest that early efficacy of fostamatinib may be a prognostic factor indicating which patients will eventually be able to discontinue therapy but continue to have a PLT in the therapeutic range.

It is also interesting to note that the two patients that relapsed after completing the T/D protocol were fully responsive to fostamatinib when therapy was reinstated. This suggests that the efficacy of fostamatinib is maintained or reinstated after discontinuation and a subsequent loss of response.

SROT, since it occurs while patients are off therapy, may not be able to be completely separated from spontaneous remission. The rate of spontaneous remission in subjects with ITP is estimated at 25–74% in the pediatric population, decreasing with age [34,35]. The reported spontaneous remission rates are lower in adults [36,37,38,39]. For some ITP treatments, the rates of SROT were higher than the rates of spontaneous remission.

Additionally, the prognostic factors for SROT and the mechanisms by which it occurs are not clear. In subjects with ITP receiving steroids as a first-line treatment, SROT rates after 6 months were close to 40% [40]. For the second-line treatment, rituximab, 20% SROT has been reported [41]. For TPO-RAs, SROT rates at six months were between 3–48% [42]. To date, no clear predictive factors for SROT have been found. In the case of TPO-RAs, achieving a response before 14 days of treatment [3] and starting treatment with romiplostim without conversion to another TPO-RA [43] are good prognostic factors for achieving SROT. Other factors, such as the type of ITP, chronicity, or previous lines of treatment, have not been validated as prognostic factors for SROT.

There are several limitations and possible sources of bias in this study. The Fostasur study was a retrospective real-world evidence study, so there was a lack of control of treatment and outcome assessments, dependence on accurate recordkeeping by others, and the potential for selection bias.

Although the number of patients with an SROT was relatively small, these findings suggest that early responses to fostamatinib may help predict which patients may be amenable to T/D while maintaining a therapeutic response. Additional studies will be required to confirm the possibility of T/D after fostamatinib treatment, to determine if predictive factors exist, and to identify possible mechanisms for SROT.

## Figures and Tables

**Table 1:** Demographics, baseline characteristics, and prior treatments for the ITP patient population treated with fostamatinib at the participating clinics in Andalusia.

	Global (n = 61)	Tapering (n = 13)	No Tapering (n = 48)	Statistics *p* Value
**Female (n, %)**	33 (54.1)	8 (61.5)	25 (52.1)	0.54
**Age at fostamatinib start, years (median, IQR)**	59 (49–78)	58 (42.5–61.5)	63 (48.5–74.5)	0.14
**ITP type: (n; %)** **Chronic** **Persistent** **Newly diagnosed**	45 (73.8) 10 (16.4) 6 (9.8)	9 (69.2) 2 (15.4) 2 (15.4)	36 (75) 8 (16.7) 4 (8.3)	0.86
**Time from diagnosis to fostamatinib, months (median, IQR)**	18 (3.8–119.8)	47 (5.5–117)	35 (8–97)	0.11
**Platelets at ITP diagnosis** **(×10^9^/L)(median, IQR)**	13 (6–24)	6.5 (2–11.5)	9.5 (3.3–23)	0.65
**Platelets at fostamatinib start (×10^9^/L)** **(median, IQR)**	16 (7.8–41.3)	15 (5–48)	20 (12–47.6)	0.24
**WHO bleeding score > 1 at fostamatinib start (n, %)**	9 (14.8)	4 (31)	5 (10.4)	0.15
**3 or more lines of treatment (n,%)** **Steroids** **Romiplostim** **Eltrombopag** **TPO-RA refractory ¨** **Rituximab** **Splenectomy**	56 (91.8) 61(100) 30 (59) 43 (70.5) 34 (66) 14 (25) 7 (15.9)	11 (84.6) 13 (100) 8 (61.5) 9 (69) 5 (38.5) 5 (38.5) 4 (30.8)	45 (93.8) 48 (100) 22 (45.9) 34 (70.8) 29 (60.4) 9 (18.8) 3 (6.3)	0.34
**Other concomitant treatment at fostamatinib start (n, %)** **Steroids** **Immunoglobulins** **Eltrombopag** **Romiplostim**	22 (23) 17 (27.9) 6 (9.8) 3 (4.9)	7 (54) 6 (46) 1 (7.7) 1 (7.7)	16 (33) 13 (27.1) 4 (8.3) 1 (2.1)	0.30
**Overall Response:(n, %)** **No response** **Response** **Complete response**	44/61 (72.1) 17/61 (27.9) 29/61 (47.5) 15/61 (24.6)	13 (100) 0 (0) 0 (0) 13 (100)	31 (64.6) 0 (0) 15 (31.3) 16 (33.3)	**0.04**
**Time to response, days (median, IQR)** **Time to complete response, days (median, IQR)**	38 (22–110) 28 (15.5–108.5)	38.5 (17.8–62.3) 28 (17.5–98.3)	40 (22–102.5) 29 (14–77)	0.09 0.18
Rescue treatment * (n, %)	15 (24.6)	2 (15.4)	13 (27.1)	0.11

IQR: interquartile range; ns: no significant difference; WHO: World Health Organization; TPO-RA: thrombopoietin receptor analogs. ¨ Platelets between 30–50 × 10^9^/L with higher doses of TPO-RA. * Rescue treatment: treatment because of relapse and necessity of acute platelet increase.

**Table 2:** Comorbidities in the patient population before starting fostamatinib (n = 61).

Comorbidity	n (%)
Hypertension	28 (45.9)
Obesity/Overweight	15 (24.6)
Dyslipidemia	14 (22.9)
Heart disease	9 (14.8)
Diabetes	8 (13.1)
Arterial thrombosis	8 (13.1)
Smoking	7 (11.5)
Overweight	7 (11.5)
Venous thrombosis	6 (9.8) *
Liver disease	2 (3.3)
Respiratory pathology	1 (1.6)
Renal insufficiency	1 (1.6)

* One distal deep vein thrombosis and two pulmonary embolisms.

## Data Availability

Data supporting this article are available from the corresponding author on request due to privacy/ethical restrictions.
